# Veno-venous extracorporeal membrane oxygenation support in the resuscitation from extreme metabolic acidosis (pH < 6.5) after drowning cardiac arrest: a case report

**DOI:** 10.1186/s12245-023-00501-4

**Published:** 2023-04-07

**Authors:** Yueyang Chai, Xinyi Zhang, Hong Liu

**Affiliations:** 1 Department of Emergency Medicine, Second Affiliated Hospital, School of Medicine, Zhejiang University, Hangzhou, 310006 China; 2 Department of Cardiology, Second Affiliated Hospital, School of Medicine, Zhejiang University, Hangzhou, 310006 China

**Keywords:** Veno-venous extracorporeal membrane oxygenation (VV-ECMO), Extreme metabolic acidosis, Drowning cardiac arrest (DCA), Targeted temperature management (TTM), Cerebral resuscitation

## Abstract

**Background:**

Resuscitation in drowning victim with cardiac arrest is difficult because of severe metabolic acidosis and multiple organ dysfunction. There is insufficient evidence to support that veno-venous extracorporeal membrane oxygenation (VV-ECMO) is beneficial for patient.

**Case presentation:**

A 44-year-old female was trapped under river when she attempted to rescue her drowning father. Furthermore, she underwent a loss of consciousness, with extreme metabolic acidosis, hypothermia and hypotension. Hence, the VV-ECMO, continuous renal replacement therapy (CRRT) and other resuscitative infusion were required. In this case, the patient did not experience any complication or neurologic deficit and reaching a complete recovery after 21 days of hospitalization.

**Conclusions:**

Our case adds further concerns in supporting a patient with extreme metabolic acidosis (pH < 6.5) and hypothermia after severe drowning cardiac arrest, including extracorporeal life support, renal support, targeted temperature management, cerebral resuscitation, etc., due to the reversible nature of this condition.

## Background

Drowning cardiac arrest (DCA) is a life-threatening disease, associated with high morbidity and mortality rates worldwide [1]. With a large amount of water aspirated into the airways, hypoxemia quickly occurs among multiple organs, including heart and brain, resulting in loss of consciousness and even cardiac arrest. And permanent neurologic impairment is found to be the most worrisome consequences in people who have been resuscitated after a drowning incident [2]. Cases of extreme metabolic acidosis and hypothermia after severe DCA were rarely reported, herein we combine veno-venous extracorporeal membrane oxygenation (VV-ECMO), continuous renal replacement therapy (CRRT) and other comprehensive supportive cares to achieve a successful treatment with favorable neurological in DCA patient with severe metabolic acidosis.

## Case presentation

A 44-year-old female presented to the emergency department of an affiliated hospital with symptom of unconsciousness. Two hours prior, she drowned and was trapped 2 m under water in a tributary near Qiantang River. Water temperature was 3 °C. She was rescued within 10 min after drowning by bystanders, without any basic life support (BLS). Then the emergency system was activated after 35 min. Arterial blood gas analysis showed pH < 6.5, lactate > 20 mmol/L, potassium 5.7 mmol/L. Cardiac arrest was immediately noticed, and then cardiopulmonary resuscitation (CPR) was performed with manual chest compressions, orotracheal intubation performed, and epinephrine administered via an intravenous access. Transient return of spontaneous circulation (ROSC) with sinus rhythm was obtained after 30 min of advanced life support (ALS). She was thus transferred on an ambulance and transported to our hospital.

**Fig. 1:**
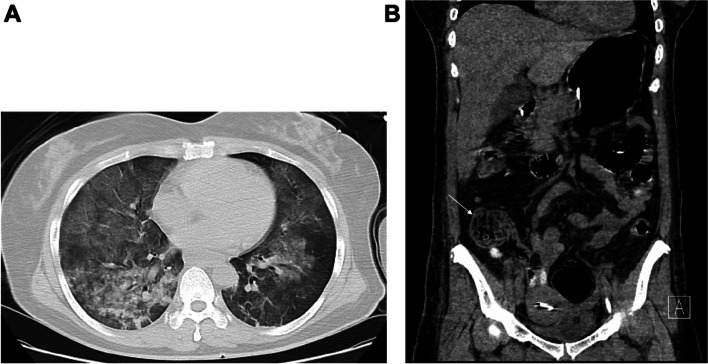
Computed tomography images.** A** Pulmonary CT revealed exudation and edema on day 1.** B** Abdominal CT revealed the edema of ascending colon on day 6

At admission, patient was directly transferred to the Emergency Intensive Care Unit (EICU). Her vital signs were unstable, indicating a hypothermia of 28.8 °C and a hypotension of 89/58 mmHg with 1.36 µg/kg/min norepinephrine maintenance. She was stuporous and Glasgow Coma Scale score was 1 + T + 1. Pupils were symmetrically equal with 3 mm diameter, but sluggish light reflex. Large amount of frothy pink sputum was aspirated from airway. Whole-body computed tomography (CT) revealed acute cerebral and pulmonary edema (Fig. 1A). Volume resuscitation and other supports were quickly performed; however, patient still remained in a multiple organ dysfunction syndrome (MODS) state with severe hypoxia, hypotension, metabolic acidosis, hyperlactatemia, gastrointestinal (GI) bleeding, anuria for more than 5 h, coagulation disorders, etc. Acute Physiology and Chronic Health Evaluation (APACHE) II score was 34. Despite maximal optimization of the ventilator settings, including a tidal volume of 480 mL (8 mL/kg), a fraction of inspired oxygen (FiO_2_) of 100%, a positive end-expiratory pressure (PEEP) of at least 15 cm H_2_O, and a driving pressure of 18 cm H_2_O, the partial arterial oxygen pressure (PaO_2_)/FiO_2_ was still less than 80 mmHg for several hours. Echocardiography showed nearly normal cardiac function with left ventricular ejection fraction > 50%. Based on that, we made a collective decision to use the VV-ECMO and CRRT. VV-ECMO was started at a flow of 4.5L/minutes, an air flow of 4L and a FiO_2_ of 100%. Once VV-ECMO was set, ventilator parameters were modified, with the tidal volume reduced to 350 mL, FiO_2_ reduced to 30%, PEEP reduced to 8 cm H_2_O, and driving pressure reduced to 12 cm H_2_O. No anticoagulation agent was used during the whole process due to GI bleeding. The hypoxia and acidosis were corrected immediately via the VV-ECMO circuit and CRRT.

Supportive care was then provided in the EICU. The ventilation and oxygenation significantly ameliorated over the first 3 days of VV-ECMO support, and she was weaned from VV-ECMO on day 6 and mechanical ventilation on day 12. She did not experience any complication or neurologic deficit in the EICU and was transferred to a rehabilitation department on day 17. Neurological status progressively improved, reaching a complete recovery after 21 days of hospitalization.

## Discussion and conclusions

According to the World Health Organization, more than 500,000 deaths each year and 0.7% of all deaths worldwide are due to unintentional drowning [1]. As the most fatal and severe consequence of drowning, DCA usually occurs within seconds to a few minutes [3]. With a large amount of water aspirated into the airways, the integrity of alveolar-capillary membrane has been disrupted and permeability highly increased, leading to reduced lung compliance, decreased pulmonary ventilation, atelectasis and bronchospasm [1]. Hence, cases of extreme metabolic acidosis and hypothermia after severe DCA were rarely reported, especially with favorable neurological outcome via VV-ECMO and comprehensive supportive cares. Based on that, this case seems remarkable for the following key points.

Firstly, early BLS and ALS are crucial to achieve ROSC and improve the prognosis of drowning victim. In our case, due to long-time DCA, patient was in MODS state, especially with severe hypoxia and metabolic acidosis. Pulmonary function deteriorated dramatically, and adequate oxygenation could only be maintained with the use of VV-ECMO. With the improvement of hypoxia and rapid infusion of crystalloid, the hemodynamic adequacy was also restored simultaneously and gradually became stable. Besides, the acid–base disorders were immediately corrected by CRRT and the internal environment was back to homeostasis.

Subsequently, cerebral resuscitation after ROSC is a big challenge in the following process. Literatures have been pointed out that the duration of drowning is highly associated with the risk of death or severe neurologic impairment after hospital discharge, approximately 88% if the time is around 11–25 min [1, 3, 4]. When the patient arrived, her body temperature was 28.8 °C. This drowning-related hypothermia could decrease the consumption of oxygen in neural cells and reduce brain metabolism, which was beneficial for the survival [5]. When she was transferred to EICU, the main goal of cerebral recovery was to establish an optimal environment via targeted temperature management (TTM). Induced hypothermia with the core temperature maintained between 35 °C and 36 °C through VV-ECMO and CRRT was conducted. Other techniques were also applied to monitor the recovery process of neurological system, such as neuron-specific enolase test, cerebral oximetry, electroencephalogram, transcranial doppler ultrasound, etc. Based on these, patient survived the peak period of cerebral edema without using mannitol.

Meanwhile, cardiac arrest from drowning is mainly due to lack of oxygen. This severe hypoxia can occur globally and even lead to MODS. Once obtaining ROSC, the restoration of circulation induces inflammation and oxidative damage in tissues, and then ischemia–reperfusion injury (IRI) immediately happens, especially in kidney and GI tract. In our scenario, patient showed anuria for 21 days. Besides, acute ischemic bowel disease remains a major therapeutic challenge. The hypoxia and inflammation prompt selective vasoconstriction of the mesenteric arterioles, at the same time the decreased venous return also impairs organ perfusion [6]. This global hypoxia and hypoperfusion states following DCA and subsequent MODS are largely involved in the onset of diffuse intestinal ischemia. Initially, the gastroscopy of patient presented stress ulcer and bleeding in the GI tract, consistence with the classical manifestation of IRI. Explosive, watery and foul-smelling stools were noted in the early stage. Classification was between type 6 and 7 based on the bristol stool chart. The absence of bowel sounds revealed the GI tract paralysis. Subsequently, the edema of GI tract occurred via abdominal CT on day 6 (Fig. 1B). The fecal cocci to bacilli ratio was also measured daily to monitor the intestinal dysbiosis. Later, fecal examination detected clostridium difficile. Based on the above symptoms and tests, fasting was performed at the beginning, along with GI tract emptying, and 250 mg vancomycin was fed via nasogastric tube every 6 h. About a week later, the condition of GI tract made some improvements, including stable bleeding and diarrhea, normal fecal cocci to bacilli ratio, and then enteral nutrition was used gradually. After 3 weeks, contrast-enhanced abdominal CT revealed the amelioration of intestinal edema, indicating the gradual recovery from the acute ischemic bowel condition.

In addition, the selection of antibiotics is another core question during the whole therapeutic procedure. To investigate the pulmonary situation, the bronchoscopy was performed and bronchoalveolar lavage fluid (BALF) was collected for metagenomic next generation sequencing (mNGS) immediately, along with culturing and sensitivity testing. Considering that the early-onset pneumonia can be due to the aspiration of polluted water, empirical therapy with broad-spectrum antibiotics (piperacillin/tazobactam + fluconazole + moxifloxacin) was started to cover the most predictable pathogens. The mNGS detected *Streptococcus dysgalactiae* for 5730 sequences, probably from the contaminated water. Then definitive therapy was substituted, with only intravenous piperacillin/tazobactam. Meanwhile, multiple parameters were monitored daily to evaluate the infectious condition of patient, including body temperature, blood inflammatory factors, culture and sensitivity testing of different specimens. The mNGS was detected again on day 6, showing *Legionella pneumophila* for 2 sequences in BALF and *Enterococcus faecium* for 1 sequence in blood. Besides, the inflammatory factors remained high level consistently. Thus, the antibiotics were switched to levofloxacin with imipenem/cilastatin to cover the pathogens. Later, the inflammatory factors decreased gradually, along with negative result of the third BALF mNGS on day 14, then the antibiotics were stopped. However, the number of white blood cell still remained high, probably associated with the ischemia of GI tract. With other supportive treatments, the patient got recovery and finally the white blood cell number returned to normal level.

DCA is a life-threatening disease, associated with high morbidity and mortality rates worldwide. Thus, high-quality CPR is required promptly after being rescued, as well as rapid evaluation and resuscitation. In the presence of severe metabolic acidosis and even MODS, multiple supportive cares, including VV-ECMO, CRRT, TTM, etc., should be quickly considered as treatment strategies for DCA since full and rapid recovery is possible. As a matter of fact, strict cooperation of emergence medical service and ECMO unit allowed this patient “back to life”.

## Data Availability

Please contact the corresponding author for all data requests.

## Abbreviations

VV-ECMO: Veno-venous extracorporeal membrane oxygenation
CRRT: Continuous renal replacement therapy
DCA: Drowning cardiac arrest
BLS: Basic life support
CPR: Cardiopulmonary resuscitation
ROSC: Return of spontaneous circulation
ALS: Advanced life support
EICU: Emergency Intensive Care Unit
CT: Computed tomography
MODS: Multiple organ dysfunction syndrome
GI: Gastrointestinal
APACHE: Acute Physiology and Chronic Health Evaluation
FiO_2_: Fraction of inspired oxygen
PaO_2_: Partial arterial oxygen pressure
TTM: Targeted temperature management
IRI: Ischemia-reperfusion injury
BALF: Bronchoalveolar lavage fluid
mNGS: Metagenomic next generation sequencing

## References

[1] Szpilman D, Bierens JJLM, Handley AJ, Orlowski JP (2012). Drowning. N Engl J Med.

[2] Schneider A, Böttiger BW, Popp E (2009). Cerebral resuscitation after cardiocirculatory arrest. Anesth Analg.

[3] Tipton MJ, Golden FSC (2011). A proposed decision-making guide for the search, rescue and resuscitation of submersion (head under) victims based on expert opinion. Resuscitation.

[4] Szpilman D, Soares M (2004). In-water resuscitation–is it worthwhile?. Resuscitation..

[5] Merchant RM, Soar J, Skrifvars MB, Silfvast T, Edelson DP, Ahmad F (2006). Therapeutic hypothermia utilization among physicians after resuscitation from cardiac arrest. Crit Care Med..

[6] Clair DG, Beach JM (2016). Mesenteric Ischemia. N Engl J Med..

